# Comparative Analysis of Codon Usage Patterns and Host Adaptation in *Merbecoviruses*

**DOI:** 10.3390/v17111479

**Published:** 2025-11-06

**Authors:** Ge Yan, Yue Li, Huimin Zhou, Giovanni Franzo, Mengdi Zheng, Hao Liu, Xiang Chen, Jianjun Dai, Wan-Ting He

**Affiliations:** 1 School of Pharmacy, China Pharmaceutical University, 639 Longmian Avenue, Nanjing 211198, China; 2 Department of Animal Medicine, Production and Health (MAPS), University of Padua, Viale dell’Università 16, 35020 Legnaro, Italy; 3 Department of Mathematics, College of Science, Nanjing Agricultural University, Nanjing 210095, China; 4 Jiangsu Key Laboratory of Zoonosis, Yangzhou University, Yangzhou 225009, China

**Keywords:** *Merbecovirus*, codon usage pattern, cross-species transmission, viral host adaptability

## Abstract

*Merbecovirus*, a subgenus of coronaviruses that includes the highly pathogenic Middle East respiratory syndrome coronavirus (MERSr-CoV), poses a significant zoonotic threat. To better understand its host adaptation and potential for cross-species transmission, we conducted a comprehensive analysis of codon usage patterns in 1967 *Merbecovirus* sequences. Phylogenetic analysis confirmed the division of *Merbecoviruses* into seven distinct clusters. Codon usage bias was found to be low and predominantly shaped by natural selection, with a consistent A/U-rich composition across the genome. Codon adaptation index (CAI) and relative codon deoptimization index (RCDI) analyses indicate that *Merbecovirus* exhibits potential host adaptation to *Sus scrofa* (pigs), *Equus caballus* (horses), and *Oryctolagus cuniculus* (rabbits), suggesting a risk of cross-species transmission. Strikingly, this genomic-level adaptation prediction is supported by emerging functional evidence: recent studies have demonstrated that key *Merbecovirus* lineages utilize diverse cell entry receptors (DPP4 or ACE2), a fundamental determinant of host tropism. For instance, the ability of the HKU5 lineage to utilize ACE2 receptors from mustelids like minks (*Neogale vison*) provides mechanistic support for the host adaptability trends inferred from our genomic analyses. By integrating existing receptor specificity data, this study provides the first systematic, large-scale analysis of codon usage across the *Merbecovirus* subgenus, elucidating key mechanisms of genomic adaptation and viral evolution. Our analytical framework provides a novel comparative perspective on host diversity and pinpoints specific surveillance priorities for mitigating future spillover risks.

## 1. Introduction

*Merbecovirus*, a subgenus of coronaviruses within the *Betacoronavirus* genus of the *Coronaviridae* family, includes a range of viruses isolated from various hosts, primarily bats, and are frequently transmitted across species and pose significant health risks to both animals and humans. Of these, Middle East respiratory syndrome coronavirus (MERS-CoV) [1], the most notable member of *Merbecovirus* that first emerged in Saudi Arabia in 2012, has since spread rapidly around the globe and caused regional and global outbreaks through cross-species transmission from dromedary camels to humans. MERS-CoV infection ranges from asymptomatic or mild respiratory illness to severe disease, including pneumonia, respiratory failure, and death, with older adults and individuals with chronic conditions at higher risk. As of September 2025, the World Health Organization (WHO) reported 2627 cumulative confirmed cases of MERS worldwide since April 2012, resulting in 947 deaths and a high fatality rate of 36% [2]. Given the serious threat that MERS-CoV poses to human health, in-depth analysis of the genomic characteristics, host range, ecological distribution, and possible public health implications of other viruses in the *Merbecovirus* subgenus could provide essential scientific knowledge for the prevention and control of future coronavirus pandemics. *Merbecovirus* subgenus includes four species, as defined by the International Committee on Taxonomy of Viruses (ICTV): *Betacoronavirus cameli*, *Betacoronavirus erinacei*, *Betacoronavirus pipistrelli*, and *Betacoronavirus tylonycteridis*. In the present classification, the four representative viruses—*Middle East respiratory syndrome-related coronaviruses* (MERSr-CoV), *Tylonycteris bat coronavirus HKU4* (Ty-BatCoV HKU4), *Pipistrellus bat coronavirus HKU5* (Pi-BatCoV HKU5), and *Hedgehog coronavirus 1* (HedCoV1)—are each assigned to one of these four species. In addition, this study included three unclassified *Merbecoviruses*, mink-derived HKU5-like viruses, *Manis javanica* HKU4-related coronavirus (MjHKU4r-CoV), and Erinaceus hedgehog coronavirus HKU31 (Ea-HedCoV HKU31), to comprehensively capture the genetic diversity within the subgenus. Of note, Ty-BatCoV HKU4 and Pi-BatCoV HKU5 were identified in Hong Kong, marking the initial detection of *Merbecovirus* five years before the MERS epidemic outbreak [3,4]. In 2024, *Pipistrellus* bat coronavirus HKU5-like viruses were further identified in two farmed minks, and the mink-derived HKU5-like CoV lineage was found to be phylogenetically closely related to viruses previously reported exclusively in bats, within which recombination events have been documented [5].

Although several hosts have been identified in *Merbecoviruses*, such as pangolins (*Manis javanica*) [6] and hedgehogs (*Erinaceus hedgehog*) [7], previous studies have demonstrated varying susceptibility to MERS-CoV among different species, with cells from rhesus monkeys (*Macaca mulatta*) [8], common marmoset (*Callithrix jacchus*) [9], goats (*Capra hircus*) [10], horses (*Equus caballus*) [11], rabbits (*Oryctolagus cuniculus*) [12], pigs (*Sus scrofa*) [13], and civets (Civettictis civetta) [14] supporting MERS-CoV replication. In addition, novel viruses similar to *Merbecovirus* and new hosts continue to be discovered, increasing the potential impact and threat posed by the *Merbecovirus* subgenus [7,15,16].

The genome of *Merbecoviruses* encodes various proteins, one main polyprotein (pp1ab), comprising, among others, the RNA-dependent RNA polymerase (RdRp), and four structural proteins: Spike (S), Envelope (E), Membrane (M), and Nucleocapsid (N). The S protein is particularly vital for invading host cells by facilitating virus attachment and cell membrane fusion [17]. The redundancy inherent in the genetic code allows for multiple codons to encode the same amino acid, a feature that influences protein production efficiency and fidelity [18]. These synonymous codons, while interchangeable, may vary in their representation within cells and in their recognition speed by ribosomes [19]. Codon selection, termed codon usage bias (CUB), is not random within or across genomes, being influenced by factors such as mutation pressure, natural selection, and environmental conditions [20,21,22]. Mutation pressure, arising from repeated nucleotide substitutions, occurs at a rate of 10^−4^–10^−5^ per nucleotide per replication cycle [23], while translation-related selection further influences codon choice. CUB can modulate viral mRNA stability, translational efficiency, and protein expression, and may facilitate immune evasion by altering RNA secondary structures or reducing CpG content [24]. Heterogeneity in CUB across genes or evolutionary stages reflects viral functional requirements and selective pressures. Understanding viral codon usage can illuminate viral evolution, gene expression regulation, and aid in vaccine development by optimizing viral protein expression for immune response generation [25,26].

Therefore, it is essential to monitor both known and potential hosts and understand the virus–host interaction, with particular attention to the host immune response. In this study, codon usage patterns of *Merbecovirus* were systematically analyzed to investigate host adaptation and to elucidate how codon usage influences viral adaptability and transmission potential.

## 2. Materials and Methods

### 2.1. Source of Target Sequences

We collected 1965 *Merbecovirus* sequences from the National Center for Biotechnology Information (NCBI) (as of the data available in April 2025, the time of this study), complemented by two additional Pipistrellus bat coronavirus HKU5-like sequences identified in farmed mink and retrieved from GenBase [27]. To guarantee the quality of the sequence, we removed sequences that were too long or too short and contained degenerate and undetermined bases (W, K, R, and X). After quality control, the five curated datasets were retained for subsequent analyses: 796 sequences for RdRp; 998 for S protein; 759 for E protein; 770 for M protein; and 815 for N protein.

To ensure consistency and comparability in further analyses, all *Merbecovirus* sequences were filtered to retain only seven lineages: MERSr-CoV, Ty-BatCoV HKU4, Pi-BatCoV HKU5, HedCoV1, Ea-HedCoV HKU31, MjHKU4r-CoV, and mink-derived HKU5-like. The virus lineages analyzed in this study were further classified according to their corresponding hosts as follows: MERSr-CoV: *Camelus dromedarius*, *Homo sapiens*, *Vespertilionidae*-MERSr; Ty-BatCoV HKU4: *Vespertilionidae*-HKU4; Pi-BatCoV HKU5: *Vespertilionidae*-HKU5; HedCoV1: *Erinaceus europaeus*; Ea-HedCoV HKU31: *Erinaceus amurensis*; MjHKU4r-CoV: *Manis javanica*; mink-derived HKU5-like coronavirus: *Neogale vison* (Supplementary Table S1). RdRp and four structural protein sequences (S, E, M, and N) were then extracted from each group for subsequent analyses.

### 2.2. Recombination Analysis

Only the main viral ORFs (RdRp and S) were selected for recombination analysis because they are homologous across the viral lineages studied and encode proteins essential to key stages of the viral life cycle, such as replication and entry. RdRp and S gene sequences were analyzed separately to identify potential recombination events using RDP4 [28]. Seven recombination detection methods implemented in RDP4 were employed: RDP [29], GENECONV [30], 3Seq [31], Chimae [32], SiScan [32], MaxChi [33], and LARD [34]. Recombination events detected by at least three methods with a *p*-value ≤ 0.05 were considered credible [35]. Recombinant sequences were subsequently removed, and the analysis was iteratively repeated until no further recombination was detected. Accordingly, five RdRp sequences and two S gene sequences were excluded from downstream analyses.

### 2.3. Phylogenetic Analysis

The sequences of RdRp and S, after recombination removal, were aligned using MAFFT v.7.520 [36] with default parameters. To minimize the influence of synonymous substitutions, phylogenetic trees were constructed based on the amino acid sequences using the maximum-likelihood (ML) method in IQ-TREE v.2.2.2.6 [37], with the best-fit substitution model selected automatically by ModelFinder Plus (MFP) option (-m MFP). Node support was assessed with ultrafast bootstrap analysis using 1000 replicates, and only nodes with bootstrap values ≥ 70% were indicated on the tree [38]. The FigTree and iTOL v.6 [39] were used to visualize and annotate phylogenetic trees.

### 2.4. Principal Component Analysis (PCA)

PCA is a multivariate statistical method used to analyze the primary trends in codon usage patterns. Sequences of all groups were recoded using 59-dimensional space of principal components (PC) that illustrated the RSCU features. Principal components PC1 and PC2, which accounted for the majority of the variance in the RSCU, were plotted; PC2 and PC3 were also analyzed to reveal secondary variation patterns and support clustering structures. PCA was performed using GraphPad Prism 9.0.

### 2.5. Nucleotide Composition Analysis

The compositional parameters of RdRp and four structural protein (S, E, M, and N) genes were calculated after removing stop codons (UAA, UGA, UAG) as well as AUG and UGG (because Met and Trp are coded by a single codon with AUG and UGG, respectively). The nucleotide frequencies of the third synonymous codon positions (A3s, G3s, C3s, U3s) were calculated using CodonW (v1.4.2). In addition, the Grand Average of Hydropathicity (Gravy) and Aromaticity (Aroma) indices were calculated using CodonW (v1.4.2) to assess the overall hydrophobicity and aromaticity of the encoded proteins, respectively. The frequencies of A, U, G, and C were calculated using CAIcal server (http://genomes.urv.es/CAIcal/; accessed on 30 September 2025). The frequencies of synonymous codons with G + C content at the first (GC1s), second (GC2s), and third (GC3s) positions were determined using the online EMBOSS (http://emboss.toulouse.inra.fr/; accessed on 30 September 2025). The G + C content at the first and second positions was combined to calculate GC12s.

### 2.6. Analysis of Relative Synonymous Codon Usage (RSCU)

RSCU refers to the relative probability of using a specific codon usage among synonymous codons encoding the same amino acid [40]. If a codon is used without preference, RSCU equals 1. Specifically, RSCU > 1 indicates a preference for the codon, RSCU ≥ 1.6 suggests a strong codon bias, indicating a high-frequency codon, and RSCU ≤ 0.6 implies a weak codon bias, indicating a low-frequency codon. RSCU values for 59 codons (excluding AUG [Met], UGG [Trp], the three stop codons UAA, UGA, and UAG, and ambiguous bases) were calculated using the CAIcal website (http://genomes.urv.es/CAIcal/; accessed on 30 September 2025). For each synonymous codon, the optimal codon was chosen based on its highest number of occurrences and largest RSCU.

Synonymous codon usage data for hosts were retrieved from the Codon Usage Database (http://codonstatsdb.unr.edu/; accessed on 30 September 2025) [41]. The RSCU values were calculated using the following formula [42]:

(1) $${RSCU}_{ij}=\frac{X_{ij}}{\sum_{j=1}^{n_{i}}X_{ij}}n_{i}\;\;$$

where *X_ij_* represents the number of occurrences of the *j*th codon for the *i*th amino acid, which has *n_i_* types of synonymous codons. Previous research demonstrated that MERS-CoV replicates in cells from rhesus monkeys, goats, horses, rabbits, pigs, civets, dromedary camels, and bats [31]. Research has shown that camels, the primary host of MERS, come into contact with wild rodents, rabbits, and possibly bats [32]. Based on this ecological and experimental context, the selection of host species for codon usage analysis in this study followed two primary criteria: biological relevance and data availability. The analysis included all confirmed natural hosts of *Merbecoviruses* with complete codon usage data available in the Codon Usage Database, namely *Homo sapiens*, *Camelus dromedarius*, *Erinaceus europaeus*, and *Manis javanica*. Potential hosts were selected based on indications from the aforementioned studies, and included pigs, horses, and rabbits, provided they possessed unique and complete entries in the database. Although bats are recognized as key reservoirs, this group was excluded from the quantitative codon adaptation analysis due to the presence of multiple, inconsistent entries at the family level in the database, which prevented a reliable and representative assessment.

Based on the computed RSCU values for five genes, heatmaps were generated using R (v4.4.3) to visually illustrate differences in codon usage patterns among different viral lineages.

### 2.7. Analysis of Dinucleotide Relative Abundance and Characterization

To understand the impact of dinucleotide frequencies on codon usage selection and identify overrepresented dinucleotides, the occurrence frequencies of all 16 possible dinucleotides within coding sequences were calculated. DAMBE software (v7.3.11) was used to compute the relative abundance of dinucleotides. The formula for calculating the dominance ratio of the 16 dinucleotides is as follows:

(2) $$P_{xy}=\frac{f_{xy}}{f_{x}f_{y}}$$

where $f_{x}$, $f_{y}$, and $f_{xy}$ denote the occurrence rate of nucleotide *X*, the prevalence of nucleotide *Y*, and the recorded occurrence frequency of the dinucleotide *XY*, respectively. When $P_{xy}$ exceeds 1.23 (or falls below 0.78), the dinucleotide *XY* is deemed as being overrepresented (or underrepresented).

### 2.8. Analysis of Effective Number of Codons (ENC)

ENC is a method used to describe the strength of preference for the usage of synonymous codons [43]. The ENC value ranges from 20 (when only one codon is used) to 61 (when all synonymous codons are equally used). A lower ENC value indicates a stronger codon preference. An ENC value below 35 suggests a strong codon preference. The ENC value can be calculated using CodonW (v1.4.2). The formula for calculating the ENC value is as follows:

(3) $$ENC=2+\frac{9}{{\bar{F}}_{2}}+\frac{1}{{\bar{F}}_{3}}+\frac{5}{{\bar{F}}_{4}}+\frac{3}{{\bar{F}}_{6}}$$

where $\bar{F}$*_k_* (*k* = 2, 3, 4, 6) represents the means of the *k*-fold degenerate amino acids, which is calculated as outlined below:

(4) $${\bar{F}}_{k}=\frac{n\sum_{j=1}^{i}{\left(\frac{n_{j}}{n}\right)}^{2}-1}{n-1}\;$$

where *n* is the total count of occurrences for the codons associated with a particular amino acid; $n_{j}$ represents the count of occurrences for the specific *j*th codon related to that amino acid.

### 2.9. ENC-GC3s Plot Analysis

ENC-GC3s plots are often used to visualize whether mutation pressure is a major determinant of codon usage bias. An ENC-GC3s plot involves constructing a scatter plot with GC3s as the independent variable and the ENC value as the dependent variable. If mutational pressure is the sole driving factor behind codon usage bias, the points on the plot will lie on a curve that can be predicted when the value of the ENC depends only on genomic composition, and computed as follows:

(5) $${ENC}_{expected}=2+GC3s+(\frac{29}{{\left(GC3s\right)}^{2}+{\left(1-GC3s\right)}^{2}})$$

Alternatively, if the points are below the standard curve, it suggests that factors other than mutational pressure influence codon usage bias. ENC values and GC3s were calculated using CodonW (v1.4.2), and GraphPad Prism 9.0 was used for plotting.

### 2.10. The Parity Rule 2 (PR2) Analysis

A PR2 plot is a method of studying the composition of codon bases. CAI website (http://genomes.urv.es/CAIcal/; accessed on 30 September 2025) was used to calculate the A3%, C3%, G3%, and T3% values. The comparison of A3/(A3 + U3) with G3/(G3 + C3) is used to assess the relationship between mutation pressure and natural selection. A = U and G = C (i.e., axis values of 0.5 and 0.5), respectively, indicating a balance between mutation pressure and natural selection.

### 2.11. Neutrality Analysis

Regression curves were computed to assess the impacts of mutational pressure and natural selection on codon usage, using the GC12s (*y*-axis) plotted against the GC3s (*x*-axis) relationship. The stronger the correlation, the closer the slope of the regression line is to 1, indicating that codon usage bias is primarily influenced by mutational pressure. Conversely, as the slope of the regression line gradually decreases (even reaching 0), it suggests an increasing role of natural selection pressure on codon usage bias. Neutrality plots were constructed by GraphPad Prism 9.0, and regression lines were calculated.

### 2.12. Correlation Analysis

To investigate the relationship between nucleotide composition and codon usage features, Spearman’s rank correlation analyses were performed in R (v4.4.3) for A3, T3, G3, C3, GC3, ENC, as well as A%, T%, G%, C%, GC%, Gravy, and Aroma values. The correlation results were then visualized as heatmaps using R (v4.4.3). To discern the primary evolutionary forces shaping codon usage bias, two analytical approaches were employed. First, the correlation between the base composition at the third codon position (A3, U3, G3, and C3) and the overall genomic base composition was assessed. A strong positive correlation, particularly between GC3 and overall GC content, was interpreted as evidence for the dominance of genome-wide mutation pressure. Second, the association between the ENC and indices of protein physicochemical properties (namely, the Gravy and Aroma indices) was evaluated. A significant association in this case was considered indicative of natural selection acting through functional constraints.

### 2.13. Codon Adaptation Index (CAI) Analysis and Relative Codon Deoptimization Index (RCDI) Analysis

CAI analysis was performed to predict the adaptability of *Merbecovirus* RdRp, S, M, N, and E genes to their natural hosts and potential hosts. The CAI value ranges from 0 to 1.0, where a higher CAI value indicates better adaptation of the virus to its host.

RCDI reflects the similarity of the codon usage between a given coding sequence and a reference genome [44]. RCDI analysis was conducted to calculate RCDI values for the encoding sequences in comparison to potential hosts. When the value is 1.0, the use of codons is appropriate for the host, and when it is greater than 1.0, the use of codons is deviated from the host. Both CAI and RCDI were computed using the CAIcal SERVER (https://ppuigbo.me/programs/CAIcal/; accessed on 30 September 2025).

## 3. Results

### 3.1. The Phylogenetic Relationship of Merbecovirus Shows Clustering Patterns Similar to Those Seen in the PCA

**Figure 1:**
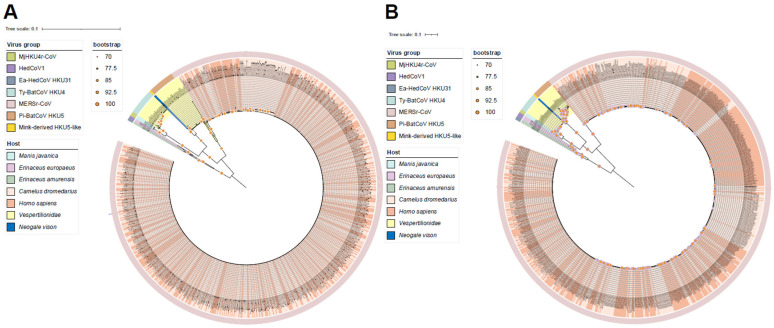
(**A**) Maximum-likelihood phylogenetic tree of *Merbecovirus* RNA-dependent RNA polymerase (RdRp) gene based on the amino acid sequences; (**B**) maximum-likelihood phylogenetic tree reconstructed based on the amino acid sequences of the spike (S) gene. Branch lengths are proportional to the number of amino acid substitutions per site, and the scale bar represents the estimated genetic distance. Branches are colored according to the host, and the outer ring indicates the virus lineages. Phylogenetic trees were constructed by IQTree based on the maximum-likelihood method with a bootstrap of 1000 replicates and visualized using the iTOL online tool (https://itol.embl.de/; accessed on 30 September 2025). Nodes exhibiting statistically significant support (bootstrap values ≥ 70%) are annotated with orange circular markers, with the marker diameter scaled proportionally to the corresponding bootstrap support value.

**Figure 2:**
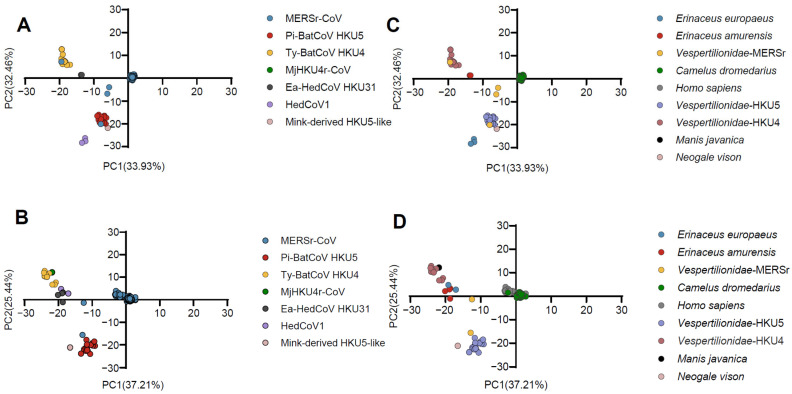
Principal Component Analysis (PCA) based on the RSCU values of 59 synonymous codons. (**A**,**B**) represent codon usage patterns of the RdRp and S gene in different *Merbecovirus* lineages, respectively; (**C**,**D**) represent codon usage clustering of the RdRp and S gene in different hosts. MERSr-CoV, Pi-BatCoV HKU5, Ty-BatCoV HKU4, MjHKU4r-CoV, Ea-HedCoV HKU31, HedCoV1, and mink-derived HKU5-like are represented in blue, red, yellow, green, dark gray, purple, and dusty pink. *Erinaceus europaeus*, *Erinaceus amurensis*, *Vespertilionidae-MERSr*, *Camelus dromedarius*, *Homo sapiens*, *Vespertilionidae-HKU5*, *Vespertilionidae-HKU4*, *Manis javanica*, and *Neogale vison* are represented in blue, red, yellow, green, light gray, purple, cameo brown, black, and dusty pink, respectively. *Vespertilionidae-MERSr*: *Vespertilionidae* carrying MERSr-CoV; *Vespertilionidae-HKU5*: *Vespertilionidae* carrying Pi-BatCoV HKU5; *Vespertilionidae-HKU4*: *Vespertilionidae* carrying Ty-BatCoV HKU4.

We performed phylogenetic analyses of *Merbecovirus* based on the RdRp gene and the S gene (Figure 1), providing an extensive understanding of the evolutionary relationships among the different viral lineages analyzed. Phylogenetic analysis revealed seven distinct host-associated groups in the *Merbecovirus* subgenus, represented by MERSr-CoV, HedCoV1, Pi-BatCoV HKU5, Ty-BatCoV HKU4, MjHKU4r-CoV, Ea-HedCoV HKU31, and mink-derived HKU5-like. Phylogenetic analysis of S and RdRp genes shows that MjHKU4r-CoV is most closely related to Ty-BatCoV HKU4, indicating a close evolutionary relationship, which is consistent with the PCA analysis where both viruses cluster together, further confirming their high genetic similarity (Figure 2, Supplementary Figure S2). In the phylogenetic tree of S and RdRp genes, virus sequences from *Homo sapiens* and *Camelus dromedarius* infected with MERSr-CoV alternate and cluster together, indicating a high degree of genomic similarity among these strains. This pattern may reflect frequent cross-host transmission events, particularly between *Homo sapiens* and *Camelus dromedarius*, which is a phenomenon that has been well-documented. Moreover, this clustering may also suggest that MERSr-CoV experiences similar selective pressures across different hosts, leading to convergent evolutionary trajectories.

The PCA plot revealed that, as observed in the phylogenetic analysis, Pi-BatCoV HKU5 and MERSr-CoV partially overlapped in the RdRp and S genes, and Ty-BatCoV HKU4 and MjHKU4r-CoV partially overlapped in the RdRp, S, and N genes (Supplementary Figures S1 and S3). At the host’s level, similarly, in the PCA plots for the RdRp, S, E, and N genes, *Vespertilionidae* from Ty-BatCoV HKU4 and *Manis javanica* from MjHKU4r-CoV showed close clustering of points. Specifically, for *Homo sapiens* and *Camelus dromedarius*, there was significant overlap in the PCA plots of all five genes, mirroring the phylogenetic relationships.

### 3.2. Nucleotide Composition Analysis Indicated a High Abundance of AU

The values of nucleotide contents in the RdRp, S, M, N, and E genes of *Merbecovirus* were analyzed (Supplementary Table S2). The results showed that nucleotides U and A were abundant in all proteins except the N protein, with about 60% AU content. Although A and C were the most abundant nucleotides in the N protein with 29.88 ± 0.36 (mean ± standard deviation) and 26.64 ± 0.38, respectively, the AU content (52.69%) was also greater than the GC content (47.31%). In addition, U3s and A3s were also higher than C3s and G3s in all proteins. For example, in RdRp, U3s (0.53 ± 0.02) and A3s (0.29 ± 0.01) were higher than C3s (0.25 ± 0.02) and G3s (0.21 ± 0.01). Similarly, the GC content in different proteins showed a consistent trend that the highest GC frequency (%) was located in position 1 and the lowest GC frequency was located in position 3. For example, the GC1/2/3 content of RdRp was 49.13%, 36.37%, and 34.41%, respectively. Analysis of five genes (RdRp, S, E, M, and N) from different host species revealed consistent AU-rich characteristics and GC positional trends across all corresponding viral genomes (Supplementary Table S3). In conclusion, the analysis of the nucleotide composition of the different proteins showed that *Merbecovirus* codons preferred U and A, and usually ended with U.

### 3.3. The RSCU of Merbecovirus Was A/U-End Biased and Opposite to the Hosts

Through RSCU analysis, the codon usage trend was studied to further understand why A/U nucleotides are preferentially used at the third position in the RdRp and four structural proteins of *Merbecovirus* (Supplementary Figures S4 and S5). In the RdRp and S protein, seven distinct viral lineages share 5 (UUU, UAU, CAU, AAU, and UGU) and 18 (UUU, AUU, GUU, UCU, CCU, GCU, UAU, CAU, CAA, AAU, GAU, GAA, CGU, GGU, UAU, AAU, UGU, and GGU) common optimal codons, respectively, all ending with A/U (Supplementary Table S4). Moreover, the over-represented codons (RSCU > 1.6) tend to be A/U-ended, while the underrepresented codons (RSCU < 0.6) are mainly G/C-ended. For different viral groups of *Merbecovirus*, there were two (UAU, CAA), three (CAU, AAU, UGU), and three (AAU, GAU, AGA) common optimal codons for E, M, and N, respectively, all ending with A/U (Supplementary Table S5). However, the optimal codons for various viral groups of *Merbecovirus* in RdRp and the four structural proteins are entirely distinct from those of all known and potential hosts (horse, rabbit, and pig), all of which exhibit a shared preference for codons ending in C/G. In addition, codons containing CpG dinucleotides (UCG, CCG, ACG, GCG, CGC, and CGA) were mostly underrepresented in RdRp and four structural proteins.

### 3.4. Mutation Pressure and Natural Selection Have Both Influenced Codon Usage Patterns

To investigate the effects of natural selection and mutational pressure on codon usage, we performed ENC-plot analysis, PR2 analysis, neutrality analysis, and correlation analysis. In this study, the ENC values for all seven groups in different proteins were above 35, indicating a low codon preference for *Merbecovirus* (Table 1). Individually, the highest ENC value was for the E (53.91 ± 4.63) and the lowest for the S (46.01 ± 4.39) coding sequence. Comparing the ENC values of seven lineages of *Merbecovirus*, we found that the highest ENC value was for MERSr-CoV (52.58 ± 4.84) and the lowest for MjHKU4r-CoV (46.30 ± 6.34).

**Figure 3:**
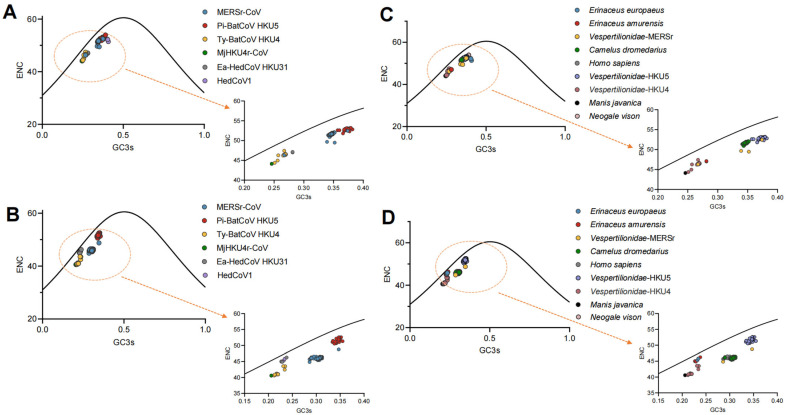
Analysis of codon usage bias using ENC-GC3s plots for the RdRp and S gene. (**A**,**B**) represent ENC plotted against GC3s for the RdRp and S gene in different *Merbecovirus* lineages, respectively; (**C**,**D**) represent ENC plotted against GC3s for the RdRp and S gene in different hosts, respectively. Solid curves represent the expected ENC values. Arrows point to the magnified views (right) of the areas marked by the dashed orange circles.

To further investigate the synonymous codon usage pattern of *Merbecovirus*, the relationship between ENC and GC3s was assessed. The ENC-GC3s plot revealed that the RdRp, S, and N (Figure 3, Supplementary Figure S6) are located below the standard curve, which indicates that natural selection could be responsible for the codon usage bias. In contrast, the M and especially E (Supplementary Figure S6) are closer to or even exceed the standard curve, suggesting that they experienced greater mutational pressure compared to other proteins. More specifically, these two proteins have been subjected to some degree of mutational pressure in MERSr-CoV and Ea-HedCoV HKU31.

**Figure 4:**
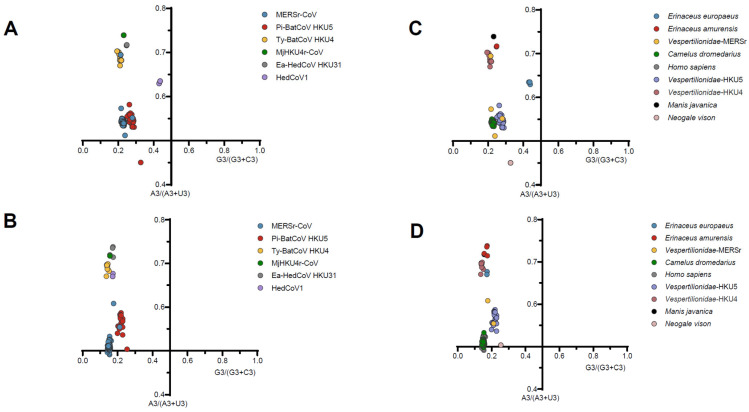
Parity Rule 2 (PR2) analysis of codon usage for the RdRp and S genes. (**A**,**B**) represent PR2 plot for the RdRp and S gene in different *Merbecovirus* lineages, respectively; (**C**,**D**) represent PR2 plot for the RdRp and S gene in different hosts, respectively. The center of the plot (0.5, 0.5), indicates the place where there is no bias in the effect of mutation or selection pressure.

**Figure 5:**
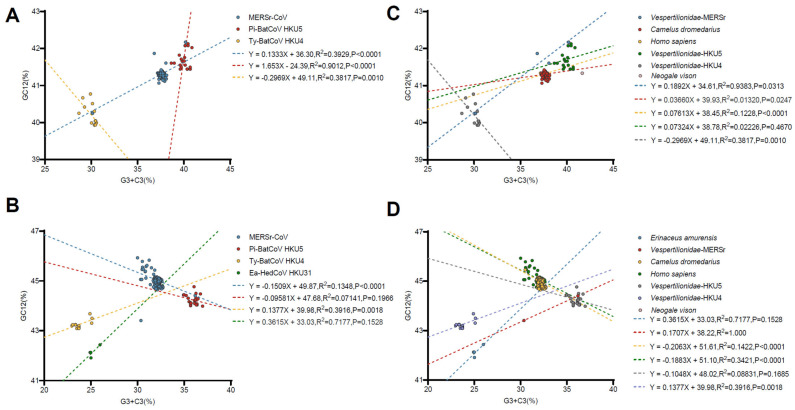
Neutrality plot analysis of codon usage (GC3s against GC12s) for the RdRp and S genes. (**A**,**B**) represent neutrality plot for the RdRp and S gene in different *Merbecovirus* lineages, respectively; (**C**,**D**) represent neutrality plot for the RdRp and S gene in different hosts, respectively. GC12s is plotted on the ordinate, and GC3s on the abscissa. The dotted line is the linear regression of GC12s against GC3s.

In addition, we performed the PR2 bias plot (Figure 4, Supplementary Figure S7) where all points are far from (0.5, 0.5) and the majority of the points are in the region of A3s/(A3s + U3s) > 0.5, G3s/(G3s + C3s) < 0.5, indicating all proteins tends to use the A/C base, further confirmation of the overlapping effect of natural selection and mutational pressure on *Merbecovirus* codon preferences. Neutrality analysis was used to further confirm whether natural selection or mutational pressure primarily shaped the codon usage patterns of *Merbecovirus*. Due to insufficient sequence data, the mink-derived HKU5-like, MjHKU4r-CoV, and HedCoV1 groups did not meet the requirements for constructing regression curves and were, therefore, excluded from the neutrality analysis. Similarly, sequences from the Ea-HedCoV HKU31 were not included in the RdRp analysis as they also failed to meet the criteria for regression curve construction. The analysis showed (Figure 5, Supplementary Figure S8) that the effect of mutation pressure on codon usage bias of RdRp in MERSr-CoV, Pi-BatCoV HKU5, and Ty-BatCoV HKU4 was only 13%, 7%, and 29%, suggesting that natural selection dominated codon usage bias in *Merbecovirus*. However, for the S protein, mutational pressure (relative to neutrality) was higher than for RdRp, as evidenced by 15%, 10%, 16%, and 36% occupancy in MERSr-CoV, Pi-BatCoV HKU5, Ty-BatCoV HKU4, and Ea-HedCoV HKU31, respectively. Although the previous ENC-plot showed that E proteins are under more mutational pressure in MERSr-CoV, neutral analysis indicated that natural selection is still dominant, namely, that natural selection contributes 72%. Correlation analysis yielded results consistent with the findings described above (Supplementary Figure S9). Combining ENC-plot, PR2 bias analysis, neutrality analysis, and correlation analysis, we conclude that despite the dominance of natural selection in codon usage bias, mutational pressure still exerts a non-negligible influence.

### 3.5. Analysis of Dinucleotide Relative Abundance and Characterization

Apart from mutation pressure and natural selection, other factors such as dinucleotide abundance are considered to influence codon usage bias. The relative abundances of the 16 dinucleotides were calculated for the RdRp and four structural proteins of *Merbecovirus* to assess their influence on codon usage selection. Deviations from the expected value (relative abundance = 1) were observed (Supplementary Figures S10 and S11), indicating non-random dinucleotide occurrences. In summary, we found four dinucleotides with high proportions (ApG, CpA, GpC, and UpC) and two dinucleotides with low proportions (CpG and GpA). Among them, CpG shows a serious underrepresentation with a mean value of only 0.59. Our investigation revealed variations in the codon usage patterns of *Merbecovirus* different-lineage dinucleotides, particularly highlighting a significant deficiency in RdRp and all structural sequences related to CpG. In the RSCU analysis, representative codons associated with CpG, except CGU encoding Arg, were underrepresented for the remaining seven codons. The CpG deficiency can be attributed to a preference for codons ending in U/A, consistent with our RSCU analysis. Overall, dinucleotide composition contributes to codon usage bias in *Merbecovirus*.

### 3.6. Codon Adaptation Index (CAI) and Relative Codon Deoptimization Index (RCDI) Analysis

To investigate the adaptability of *Merbecovirus* to its natural and potential host, we calculated CAI and RCDI values for eight reported hosts and three potential hosts of *Merbecovirus*, respectively (Table 2). CAI represents the relationship between gene expression levels and codon usage patterns, and higher CAI values indicate stronger adaptability. RCDI represents the adaptation degree of a pathogen to its host species, with a lower RCDI value meaning higher adaptation. It was found that the CAI and RCDI scores of different hosts showed diverse adaptive patterns, and that CAI as well as RCDI varied for different proteins in the same host. For instance, our analysis revealed that the average CAI score (0.71 ± 0.02) and RCDI score (1.48 ± 0.17) for *Homo sapiens* were comparatively higher than those for other hosts, suggesting that *Merbecovirus* is better adapted to humans. Specifically, the RdRp protein exhibited improved adaptation in humans, as indicated by a higher CAI (0.73) and lower RCDI (1.36). Based on CAI and RCDI analyses, pigs, horses, and rabbits exhibited codon adaptation patterns similar to those observed in known natural hosts. Interestingly, among the potential hosts, horses displayed the highest average CAI (0.64 ± 0.02) and RCDI (1.6 ± 0.22) scores, possibly indicating a favorable codon adaptation of *Merbecovirus* in horse cells. 

## 4. Discussion

The potential threat posed by *Merbecovirus* is underscored by its ability to cross species barriers, infecting various mammalian hosts and raising concerns about its capacity to cause future zoonotic outbreaks. Phylogenetic analyses suggest that *Merbecovirus* transmitted from bats or wild animals to farmed animals or humans have different geographical origins, implying the existence of several animal hosts [45]. A comprehensive codon usage analysis of RdRp and the four structural proteins (S, E, M, and N) in *Merbecoviruses* enables a comparative evaluation of their molecular evolution and host adaptability. Importantly, this prediction finds strong support in a growing body of in vitro and in vivo evidence regarding the fundamental mechanism of viral entry: receptor specificity.

Systematic analyses of nucleotide composition revealed that *Merbecoviruses* exhibit a high abundance of A and U, particularly at the third codon position. RSCU analysis further showed that *Merbecoviruses* preferentially use A/U-ending codons. This preference is not a virus-specific adaptation but rather a common molecular feature of RNA viruses (also observed in MERS-CoV, SARS-CoV-2, and various mammalian RNA viruses) whose coding sequences typically exhibit a compositional bias characterized by reduced C/G-ending and enriched A/U-ending codons [46,47,48,49,50,51]. A shared AU-rich bias was observed in both the viruses and their primary bat hosts, with host genomic GC content ranging from 36% to 50%. Given the AT-rich nature of the human genome (58%), such nucleotide composition likely facilitates viral adaptability and evolution within host environments [52,53,54,55]. Studies show that the zinc-finger antiviral protein (ZAP) binds CpG-rich viral RNA to suppress replication [56]; conversely, low CpG content in viral RNA can help evade immune recognition by receptors like RIG-I, delaying interferon production and facilitating viral escape [57]. Although dinucleotide composition reflects viral family characteristics rather than host genomes, our results show consistently low viral CpG levels across different hosts. Moreover, the RSCU pattern of *Merbecoviruses* correlates poorly with that of the host, which may reduce translational efficiency but simultaneously support the correct folding of viral proteins [58,59].

The ENC values for all viral groups were consistently above 35, indicating a generally low codon usage bias, which may facilitate efficient replication, transcription, and translation during host infection [40]. Among them, MERSr-CoV exhibited the highest ENC values, suggesting particularly weak codon bias that could reflect distinct codon preferences and support efficient replication in vertebrate hosts. Notably, the E and M proteins of MERSr-CoV from *Camelus dromedarius* and *Homo sapiens*, as well as partial sequences of Ea-HedCoV HKU31, deviated above the standard curve, with the E protein showing the greatest deviation—a pattern also reported in recent MERS-CoV studies—indicating potential selective pressures and accelerated evolution that may affect pathogenicity and immune evasion [60,61]. Correlation analyses, PR2, and neutrality analyses further revealed that natural selection predominantly shapes codon usage bias, with mutational pressure contributing to a lesser extent. Recent comparative analyses of codon usage patterns across *Betacoronaviruses* suggest that both mutational pressure and natural selection contribute to shaping their evolutionary trajectories. *Embecoviruses* (e.g., HCoV-OC43 and HCoV-HKU1) exhibit codon usage profiles indicative of long-term adaptation to human hosts, characterized by relatively weak bias predominantly governed by mutational pressure [62]. In contrast, *Sarbecoviruses*, particularly SARS-CoV-2, display progressive optimization toward human-preferred codons over the course of ongoing evolution [63,64].

The canonical receptor for MERS-CoV is human dipeptidyl peptidase-4 (hDPP4) [65]. In the phylogenetic analysis of S and RdRp genes, our finding that MERSr-CoV sequences from humans and camels cluster closely together is consistent with this known receptor usage as camels are the established zoonotic reservoir. Furthermore, the recently identified pangolin-derived MjHKU4r-CoV, which clusters with bat HKU4 viruses in our phylogenetic and PCA analyses, has also been experimentally proven to utilize hDPP4 for efficient cell entry, confirming its potential for zoonotic transmission [6].

Perhaps the most significant validation of our codon-based predictions comes from the recent paradigm shift in understanding *Merbecovirus* receptor usage. Bat-origin *Merbecoviruses*, such as NeoCoV, PDF-2180, MOW15-22, and PnNL 2018B, have been confirmed to use ACE2 as a functional receptor [66,67]. It is now established that several *Merbecovirus* lineages have evolved to use angiotensin-converting enzyme 2 (ACE2) instead of DPP4. Notably, the bat virus NeoCoV and its close relative PDF-2180 can utilize ACE2 orthologs from various bat species for entry [66]. More directly relevant to our findings, the HKU5 lineage—viruses from which were identified in farmed minks in our dataset—has been demonstrated to use ACE2 from its natural bat host (*Pipistrellus abramus*) and, crucially, from American mink (*Neogale vison*) [68,69]. These viruses exhibit distinct receptor usage due to significant receptor-binding domain (RBD) sequence divergence. NeoCoV/PDF-2180 retains a MERS-CoV-like RBD fold but forms a more compact ACE2-binding interface via conformational changes, relying on glycosylation sites N54 and N329, unlike SARS-CoV-2 or NL63 [66,70,71,72,73]. Receptor shift results from key amino acid changes and RBD domain recombination, with different lineages evolving independently: NeoCoV/PDF-2180 uses bat ACE2, while HKU5-CoV-2 employs a novel interface for human ACE2, illustrating convergent evolution [66,74]. Therefore, codon usage bias in viral genomes appears to be associated with the evolution of receptor-binding domains, suggesting a plausible direction for exploring viral host adaptation mechanisms.

Analysis of CAI and RCDI revealed that *Merbecoviruses* exhibit codon adaptation patterns in pigs, horses, and rabbits similar to those observed in their natural hosts. These findings are consistent with in vitro experiments and animal infection studies, in which MERSr-CoV has been shown to complete its replication cycle and generate infectious viral particles in primary cells or in vivo models of these species [12,13,75]. Together, the concordance between codon adaptation metrics and experimental infection data provides multi-dimensional evidence supporting the role of pigs, horses, and rabbits as potential susceptible hosts of *Merbecoviruses*, warranting further investigation into their potential involvement in cross-species viral transmission.

In conclusion, our comprehensive codon usage analysis reveals the evolutionary adaptation of *Merbecoviruses* to a diverse range of hosts. The agreement between our genomic predictions and established functional studies on receptor tropism underscores the reliability of this approach. The identification of potential new host species, coupled with the demonstrated ability of certain *Merbecovirus* lineages to utilize different entry receptors—a trait potentially acquired through recombination events—significantly expands the perceived host range of these viruses and highlights an ongoing risk of cross-species transmission.

## Figures and Tables

**Table 1:** The effective number of codons (ENC) values for viral structural protein (S, E, M, N) and nonstructural gene (RdRp) of different *Merbecovirus* lineages.

Species	ENC					
	RDRP	S	E	M	N	AVERAGE
HedCoV1	52	45.65	53.42	42.69	49.71	48.69 ± 3.99
Ea-HedCoV HKU31	47.06	45.29	50.15	48.56	51.85	48.58 ± 2.29
Ty-BatCoV HKU4	46.36	41.26	51.09	47.17	48.81	46.94 ± 3.27
Pi-BatCoV HKU5	52.80	51.62	47.90	53.34	52.00	51.53 ± 1.91
MERSr-CoV	51.60	46.04	55.91	59.85	49.50	52.58 ± 4.84
MjHKU4r-CoV	44.12	40.60	57.90	41.11	47.77	46.30 ± 6.34
Mink-derived HKU5-like	53.93	51.62	61.00	58.47	51.22	55.24 ± 4.31

**Table 2:** The codon adaptation index (CAI) and the relative codon deoptimization index (RCDI) of natural hosts and potential hosts of *Merbecovirus*.

(**a**)											
	***Bos taurus***	***Camelus dromedarius***	***Equus asinus***	***Erinaceus europaeus***	***Capra hircus***	***Homo sapiens***	***Ovis aries***	***Rhinolophus ferrumequinum***	***Sus scrofa***	***Equus caballus***	***Oryctolagus cuniculus***
RdRp	0.65	0.59	0.64	0.67	0.66	0.73	0.61	0.52	0.61	0.65	0.56
S	0.63	0.57	0.61	0.65	0.64	0.71	0.59	0.49	0.58	0.63	0.53
E	0.61	0.57	0.58	0.66	0.61	0.68	0.57	0.47	0.57	0.61	0.53
M	0.62	0.57	0.59	0.61	0.62	0.69	0.58	0.48	0.58	0.62	0.54
N	0.66	0.61	0.66	0.71	0.67	0.73	0.64	0.51	0.62	0.67	0.57
AVERAGE	0.64 ± 0.02	0.58 ± 0.02	0.62 ± 0.03	0.66 ± 0.03	0.64 ± 0.02	0.71 ± 0.02	0.6 ± 0.02	0.49 ± 0.02	0.59 ± 0.02	0.64 ± 0.02	0.55 ± 0.02
(**b**)											
	***Bos taurus***	***Camelus dromedarius***	***Equus asinus***	***Erinaceus europaeus***	***Capra hircus***	***Homo sapiens***	***Ovis aries***	***Rhinolophus ferrumequinum***	***Sus scrofa***	***Equus caballus***	***Oryctolagus cuniculus***
RdRp	1.45	1.56	1.49	1.51	1.42	1.36	1.54	1.82	1.54	1.45	1.65
S	1.65	1.78	1.72	1.73	1.61	1.53	1.8	2.06	1.77	1.64	1.89
E	1.94	2.18	2.2	1.89	1.99	1.79	2.19	3.03	2.1	2.01	2.24
M	1.44	1.52	1.62	1.56	1.43	1.35	1.61	1.79	1.52	1.45	1.61
N	1.48	1.53	1.46	1.38	1.43	1.39	1.53	1.8	1.55	1.45	1.65
AVERAGE	1.59 ± 0.19	1.71 ± 0.25	1.7 ± 0.27	1.61 ± 0.18	1.58 ± 0.22	1.48 ± 0.17	1.73 ± 0.25	2.1 ± 0.48	1.7 ± 0.22	1.6 ± 0.22	1.81 ± 0.24

## Data Availability

The original contributions presented in the study are included in the article/Supplementary Material. Further inquiries can be directed to the corresponding author.

## Supplementary Materials

The following supporting information can be downloaded at https://www.mdpi.com/article/10.3390/v17111479/s1, Figure S1: Principal Component Analysis (PCA) based on the RSCU values of 59 synonymous codons. Scatter plot of Merbecovirus genes on the plane defined by the first two principal components (PC1 and PC2); Figure S2: Principal Component Analysis (PCA) of major Merbecovirus genes based on the second and third principal components (PC2 and PC3); Figure S3: Principal Component Analysis (PCA) of major Merbecovirus genes based on the second and third principal components (PC2 and PC3); Figure S4: Heatmap of relative synonymous codon usage (RSCU) values for RdRp gene and S gene; Figure S5: Heatmap of relative synonymous codon usage (RSCU) values for E gene, M gene, and N gene; Figure S6: ENC-GC3s plot analysis of codon usage for the structural genes E, M, and N; Figure S7: Parity Rule 2 (PR2) analysis of codon usage for the E, M, and N genes; Figure S8: Neutrality plot analysis of codon usage (GC3s against GC12s) for the E, M, and N genes; Figure S9: Correlations among nucleotide composition and amino acid usage features of Merbecovirus; Figure S10: Relative abundance analysis of 16 dinucleotides in the RdRp and S genes; Figure S11: Relative abundance analysis of 16 dinucleotides in the E, M, and N genes; Table S1: Overview of Merbecovirus seven lineages and their associated host-based groups; Table S2: The results of nucleotide composition analysis in viral structural protein (S, E, M, N) and nonstructural gene (RdRp) of Merbecovirus; Table S3: The results of nucleotide composition analysis in viral structural protein (S, E, M, N) and nonstructural gene (RdRp) of host-based groups of Merbecovirus; Table S4: The relative synonymous codon usage (RSCU) patterns of 59 codons encoding 18 amino acids in RdRp and S for various lineages of Merbecovirus, compared with known and potential hosts; Table S5: The relative synonymous codon usage (RSCU) patterns in M, N, and E proteins for various lineages of Merbecovirus, compared with known and potential hosts.
