# Supplementary material for: Comparative Analysis of Codon Usage Patterns and Host Adaptation in Merbecoviruses
Source: Viruses. 2025 Nov 6;17(11):1479. doi: 10.3390/v17111479 (PMC12656749; doi:10.3390/v17111479)
Supplement: Supplementary file 1 [file viruses-17-01479-s001.zip › Supplementary Figure.pdf]

Supplementary Figure 1

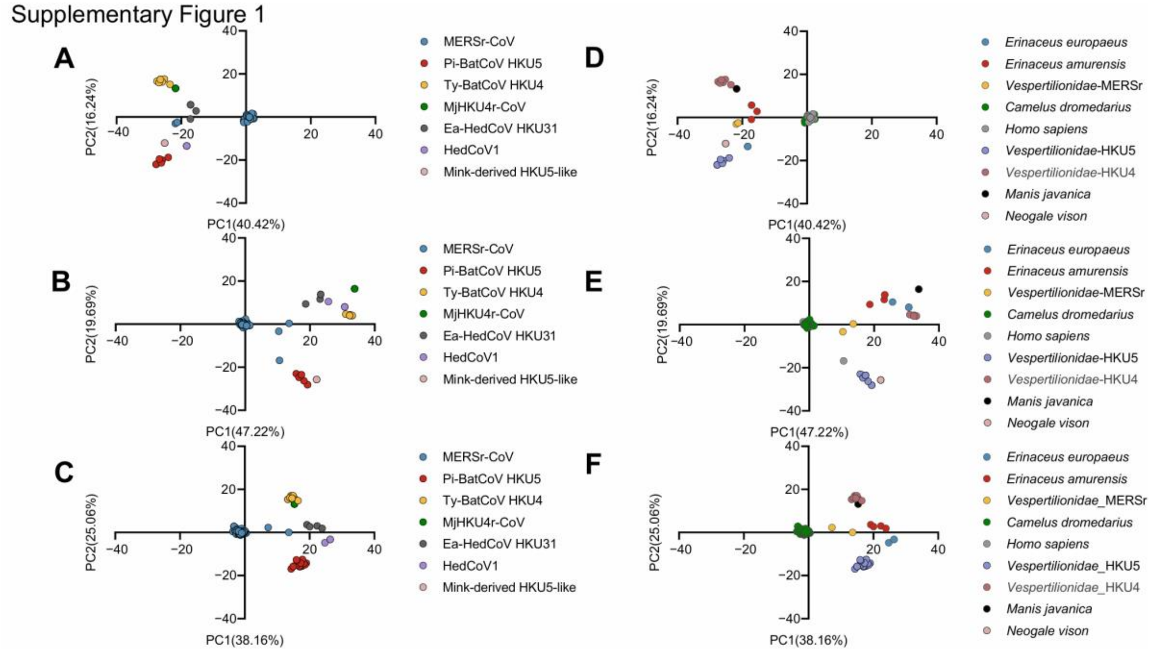

Figure S1: Principal Component Analysis (PCA) based on the RSCU values of 59 synonymous codons. Scatter plot of *Merbecovirus* genes on the plane defined by the first two principal components (PC1 and PC2). (A–C) represent the PCA of structural genes E, M, and N in different *Merbecovirus* lineages, respectively; (D–F) represent the PCA of structural genes E, M, and N in different hosts, respectively. MERSr-CoV, Pi-BatCoV HKU5, Ty-BatCoV HKU4, MjHKU4r-CoV, Ea-HedCoV HKU31, HedCoV1, and mink-derived HKU5-like are represented in blue, red, yellow, green, dark gray, purple, and dusty pink. *Erinaceus europaeus*, *Erinaceus amurensis*, *Vespertilionidae-MERSr*, *Camelus dromedarius*, *Homo sapiens*, *Vespertilionidae-HKU5*, *Vespertilionidae-HKU4*, *Manis javanica*, and *Neogale vison* are represented in blue, red, yellow, green, light gray, purple, cameo brown, black, and dusty pink, respectively. *Vespertilionidae-MERSr*: *Vespertilionidae* carrying MERSr-CoV; *Vespertilionidae-HKU5*: *Vespertilionidae* carrying Pi-BatCoV HKU5; *Vespertilionidae-HKU4*: *Vespertilionidae* carrying Ty-BatCoV HKU4.

Supplementary Figure 2

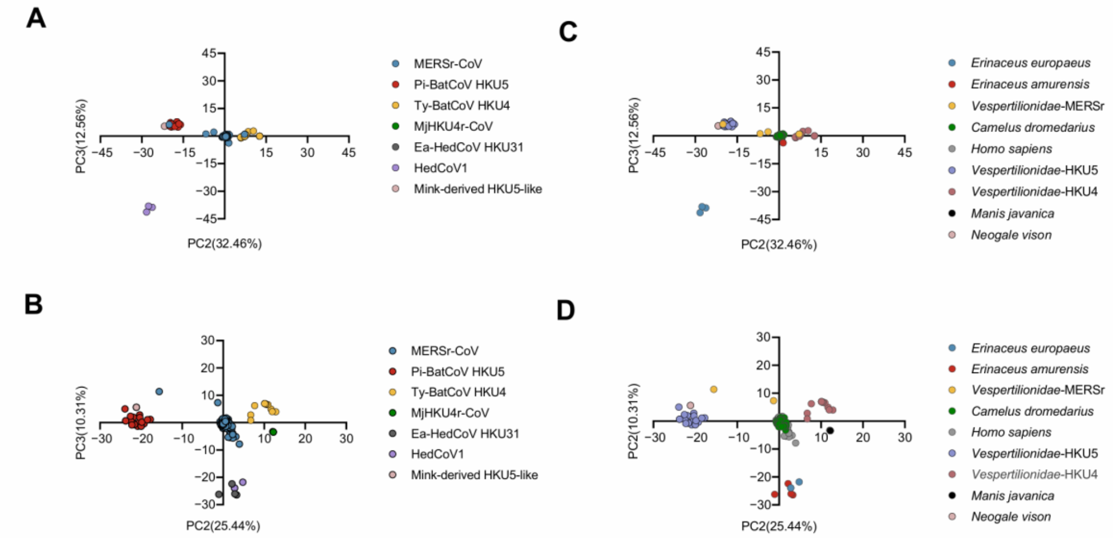

Figure S2: Principal Component Analysis (PCA) of major *Merbecovirus* genes based on the second and third principal components (PC2 and PC3). (A,B) represent codon usage patterns of the RdRp and S gene in different *Merbecovirus* lineages, respectively; (C,D) represent codon usage clustering of the RdRp and S gene in different hosts.

Supplementary Figure 3

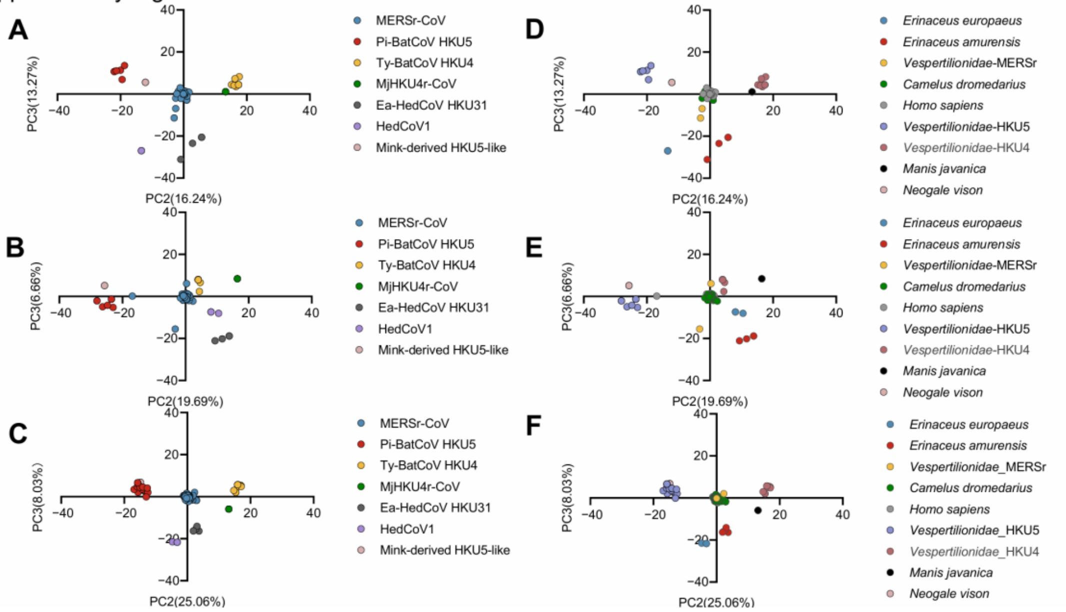

Figure S3: Principal Component Analysis (PCA) of major *Merbecovirus* genes based on the second and third principal components (PC2 and PC3). (A–C) represent the PCA of structural genes E, M, and N in different *Merbecovirus* lineages, respectively; (D–F) represent the PCA of structural genes E, M, and N in different hosts, respectively.

Supplementary Figure 4

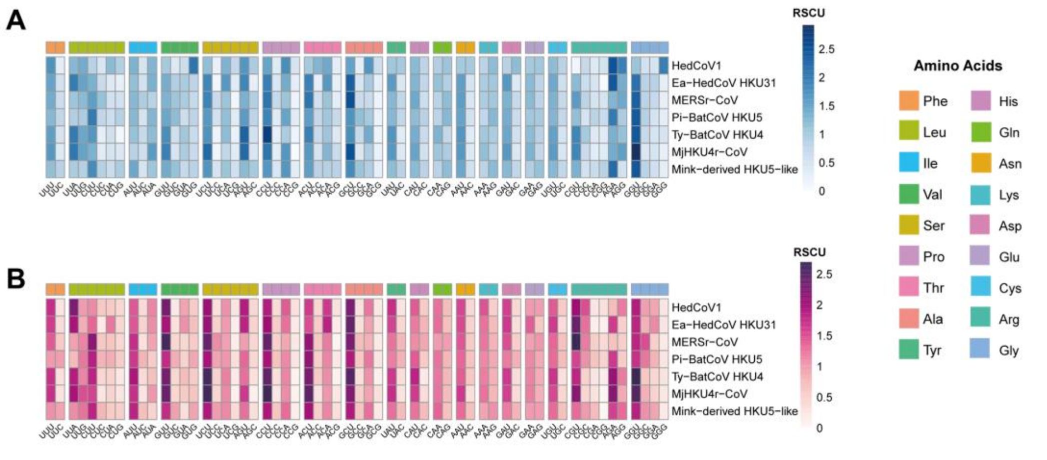

Figure S4: Heatmap of relative synonymous codon usage (RSCU) values for (A) RdRp gene and (B) S gene. In each panel, rows represent the 59 synonymous codons, and columns represent different viral lineages.

Supplementary Figure 5

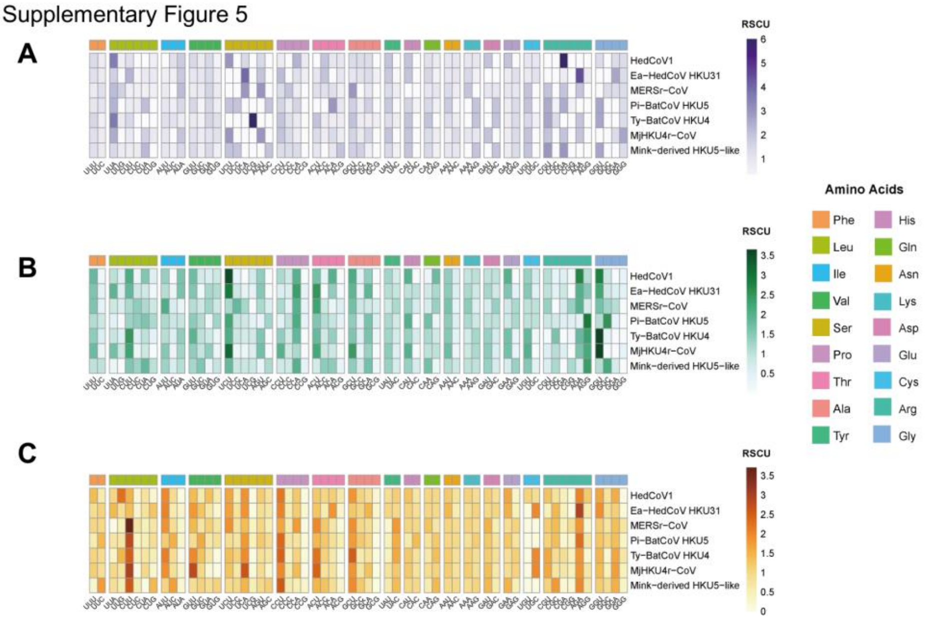

Figure S5: Heatmap of relative synonymous codon usage (RSCU) values for (A) E gene, (B) M gene, and (C) N gene. In each panel, rows represent the 59 synonymous codons, and columns represent different viral lineages.

Supplementary Figure 6

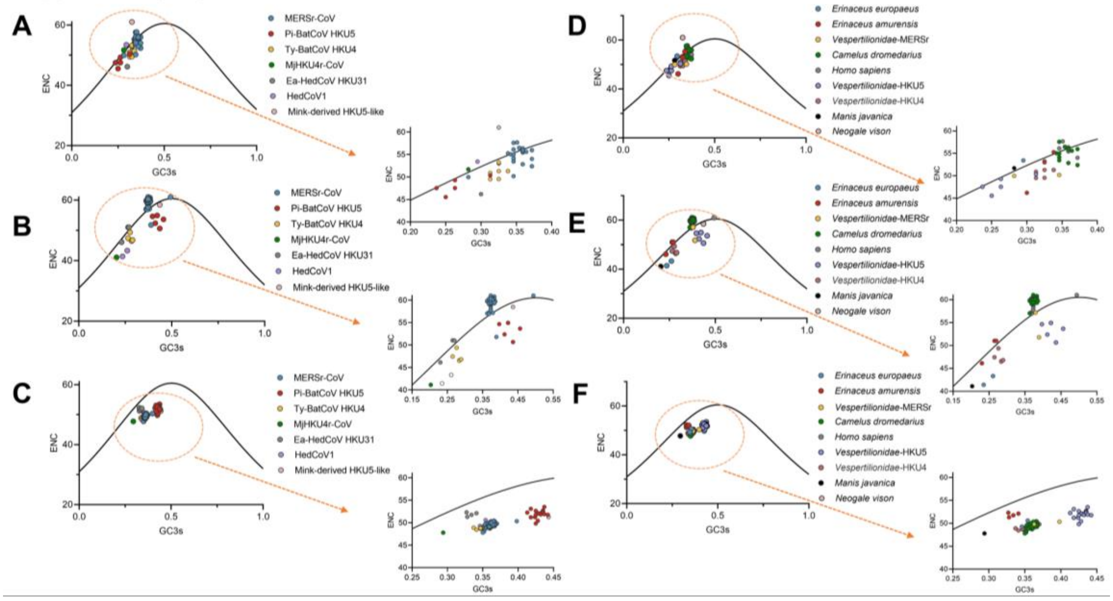

Figure S6: ENC-GC3s plot analysis of codon usage for the structural genes E, M, and N. (A–C) represent the ENC plotted against GC3s of structural genes E, M, and N in different *Merbecovirus* lineages, respectively; (D–F) represent the ENC plotted against GC3s of structural genes E, M, and N in different hosts, respectively. Solid curves represent the expected ENC value.

Supplementary Figure 7

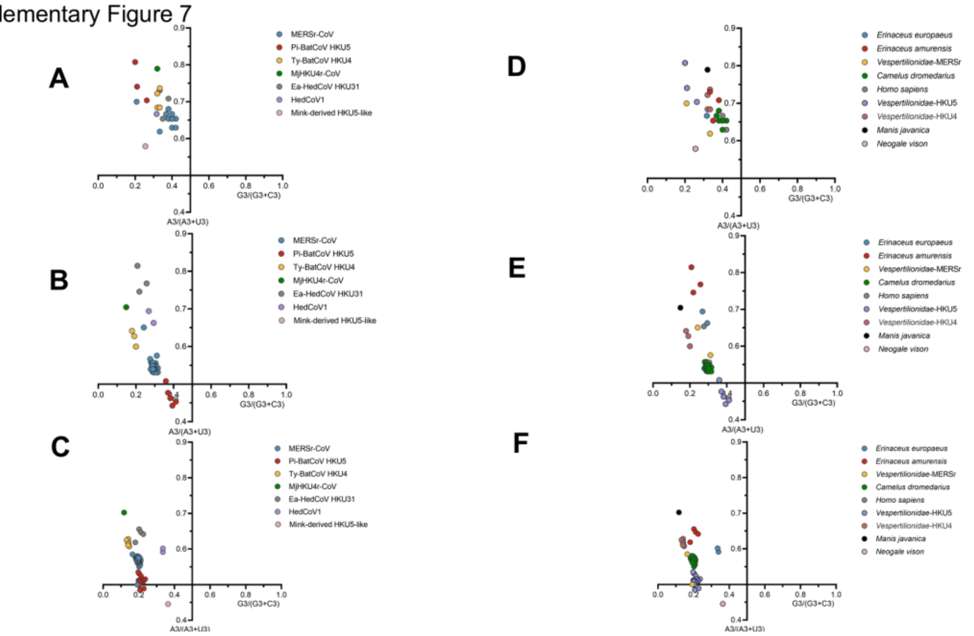

Figure S7: Parity Rule 2 (PR2) analysis of codon usage for the E, M, and N genes. (A–C) represent the PR2 plot of structural genes E, M, and N in different *Merbecovirus* lineages, respectively; (D–F) represent the PR2 plot of structural genes E, M, and N in different hosts, respectively. The center of each plot (0.5, 0.5) indicates the position where there is no bias in the effect of mutation or selection pressure.

Supplementary Figure 8

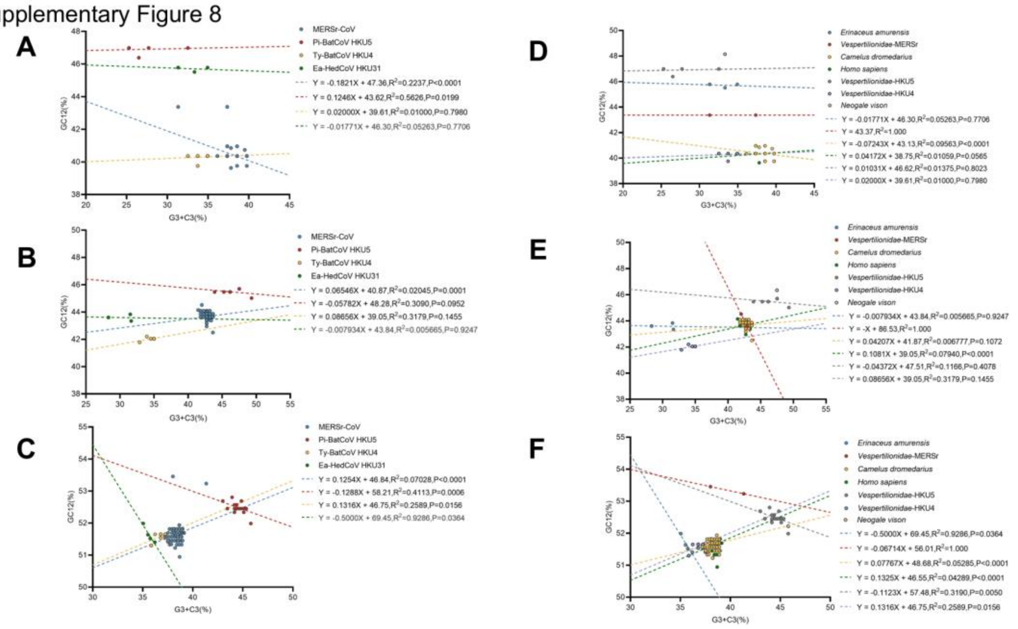

Figure S8: Neutrality plot analysis of codon usage (GC3s against GC12s) for the E, M, and N genes. (A–C) represent the neutrality plot of structural genes E, M, and N in different *Merbecovirus* lineages, respectively; (D–F) represent the neutrality plot of structural genes E, M, and N in different hosts, respectively. GC12s is plotted on the ordinate, and GC3s on the abscissa.

Supplementary Figure 9

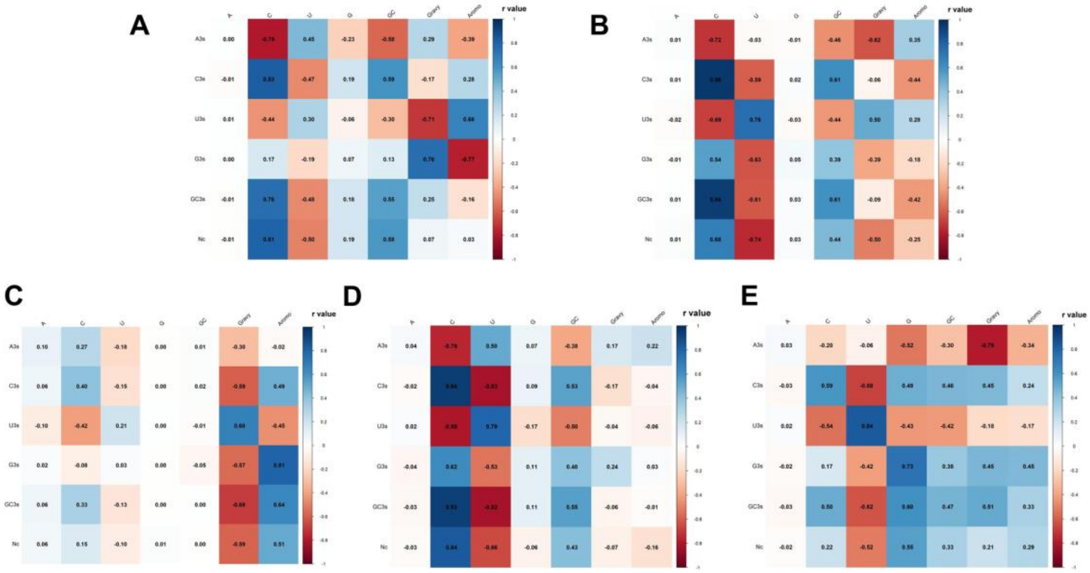

Figure S9: Correlations among nucleotide composition and amino acid usage features of *Merbecovirus*. The heatmap depicts correlation coefficients for A3, T3, G3, C3, GC3, ENC, A%, T%, G%, C%, GC%, Gravy, and Aroma values of the RdRp (A) and structural S(B), E(C), M(D), and N(E).

Supplementary Figure 10

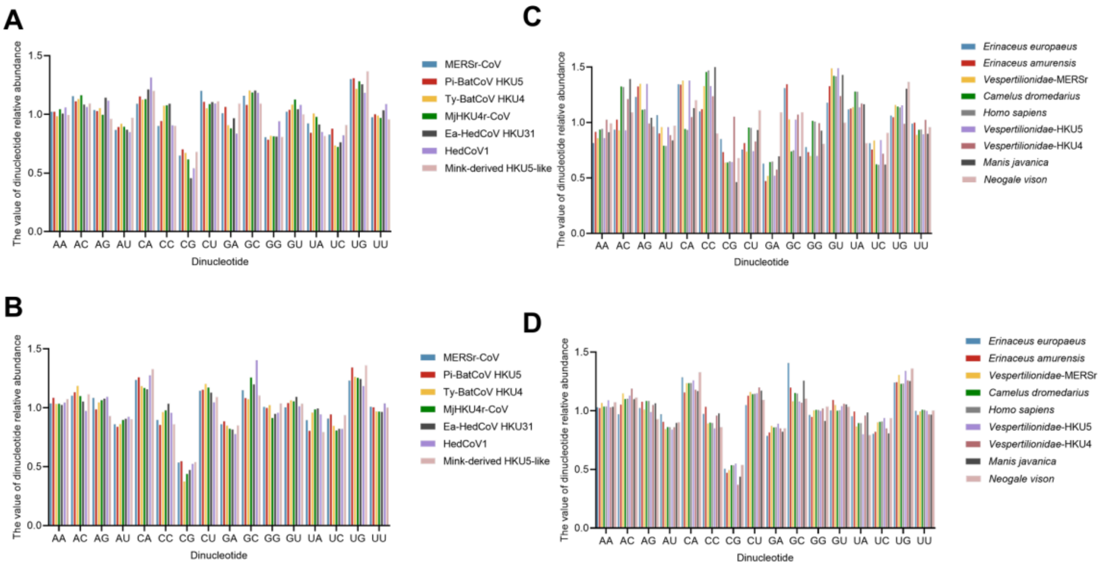

Figure S10: Relative abundance analysis of 16 dinucleotides in the RdRp and S genes. (A,B) represent dinucleotide relative abundance for the RdRp and S gene in different *Merbecovirus* lineages, respectively; (C,D) represent dinucleotide relative abundance for the RdRp and S gene in different hosts, respectively.

Supplementary Figure 11

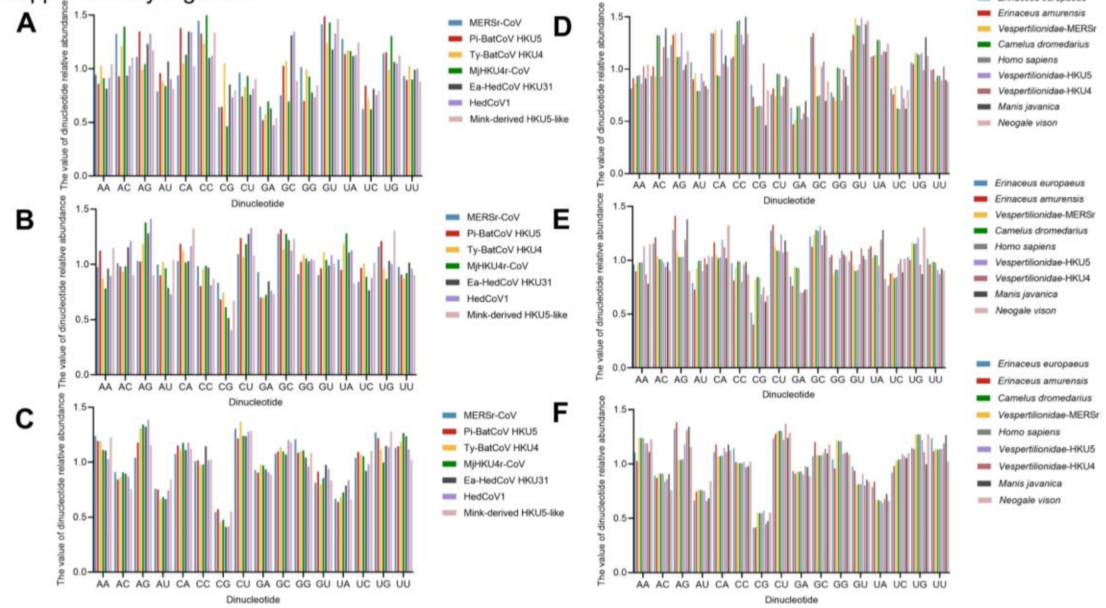

Figure S11: Relative abundance analysis of 16 dinucleotides in the E, M, and N genes. (A–C) represent the dinucleotide relative abundance of structural genes E, M, and N in different *Merbecovirus* lineages, respectively; (D–F) represent the dinucleotide relative abundance of structural genes E, M, and N in different hosts, respectively.
